# Quality and cost of healthcare services in patients with diabetes in Iran: Results of a nationwide short-term longitudinal survey

**DOI:** 10.3389/fendo.2023.1099464

**Published:** 2023-03-16

**Authors:** Mohsen Abbasi-Kangevari, Farnam Mohebi, Seyyed-Hadi Ghamari, Mitra Modirian, Nazila Shahbal, Naser Ahmadi, Yosef Farzi, Mehrdad Azmin, Shahin Roshani, Hossein Zokaei, Maryam Khezrian, Shahedeh Seyfi, Mohammad Keykhaei, Fatemeh Gorgani, Saral Rahimi, Negar Rezaei, Shahab Khatibzadeh, Saeid Shahraz

**Affiliations:** ^1^ Non-Communicable Diseases Research Center, Endocrinology and Metabolism Population Sciences Institute, Tehran University of Medical Sciences, Tehran, Iran; ^2^ Haas School of Business, University of California Berkeley, Berkeley, CA, United States; ^3^ The Netherlands Cancer Institute (NKI), Amsterdam, Netherlands; ^4^ Feinberg Cardiovascular and Renal Research Institute, Northwestern University, School of Medicine, Chicago, IL, United States; ^5^ Endocrinology and Metabolism Research Center, Endocrinology and Metabolism Clinical Sciences Institute, Tehran University of Medical Sciences, Tehran, Iran; ^6^ Heller School for Social Policy and Management, Brandeis University, Waltham, MA, United States; ^7^ Institute for Clinical Research and Health Policy Studies, Tufts Medical Center, Boston, MA, United States

**Keywords:** patient care continuity, diabetes mellitus, healthcare utilization, patient journey, care quality, care standard

## Abstract

**Aims:**

To investigate the journey of patients with diabetes in the healthcare system using nationally-representative patient-reported data.

**Methods:**

Participants were recruited using a machine-learning-based sampling method based on healthcare structures and medical outcome data and were followed up for three months. We assessed the resource utilization, direct/indirect costs, and quality of healthcare services.

**Results:**

One hundred fifty-eight patients with diabetes participated. The most utilized services were medication purchases (276 times monthly) and outpatient visits (231 times monthly). During the previous year, 90% of respondents had a laboratory fasting blood glucose assessment; however, less than 70% reported a quarterly follow-up physician visit. Only 43% had been asked about any hypoglycemia episodes by their physician. Less than 45% of respondents had been trained for hypoglycemia self-management. The annual average health-related direct cost of a patient with diabetes was 769 USD. The average out-of-pocket share of direct costs was 601 USD (78.15%). Medication purchases, inpatient services, and outpatient services summed up 79.77% of direct costs with a mean of 613 USD.

**Conclusion:**

Healthcare services focused solely on glycemic control and the continuity of services for diabetes control was insufficient. Medication purchases, and inpatient and outpatient services imposed the most out-of-pocket costs.

## Introduction

Diabetes mellitus (hereafter diabetes) is one of the most significant global public health concerns causing 916 Disability-Adjusted Life-Years (DALYs) per 100,000 population in 2019 worldwide (1). Despite the previous efforts, diabetes remains the second most significant cause of reduced healthy life expectancy (2). The International Diabetes Federation (IDF) estimated that unless effective prevention methods are employed, the prevalence of diabetes will increase by 54% in 2045 (3). On the national scale, the prevalence of diabetes has risen roughly 30% in Iran in the last decade, which is alarmingly higher than the global estimations (4).

The presence of diabetes is associated with increased mortality from infections, cardiovascular diseases, stroke, chronic kidney diseases, chronic liver diseases, and cancers (5). Uncontrolled diabetes can impose high direct and indirect costs on patients and healthcare systems. The annual costs attributable to diabetes were estimated to be US$1.31 trillion worldwide or 1.8% of the global gross domestic product (GDP), two-thirds of which were direct medical costs, and the one-third were indirect costs (6). In the meantime, diabetes costs are expected to grow considerably, disproportionately affecting low- and middle-income countries, where 80% of patients with diabetes live (7).

Delivery of essential medications, blood glucose management, cardiometabolic risk factors elimination, and early screening for complications *via* well-organized care reduce acute and chronic complications and extends healthy life expectancy among patients with diabetes (8, 9). Nevertheless, the comprehensive, evidence-based diabetes care delivery is suboptimal even in well-resourced health systems (10). Notably, multicomponent quality improvement programs have been beneficial in achieving diabetes care goals (11). In this sense, investigating healthcare quality and costs for diabetes is essential to make evidence-based decisions to lower the costs and increase the quality of care.

Thus, the objective of this study was to investigate the journey of patients with diabetes in the healthcare system *via* assessing the healthcare utilization, quality, and costs on a national level in Iran based on the results of the Iran Quality of Care in Medicine Program (IQCAMP). IQCAMP is a series of longitudinal surveys focusing on seven high-prevalence and high-cost diseases, including chronic obstructive pulmonary disease, congestive heart failure, diabetes mellitus, end-stage renal failure, major depressive disorder, myocardial infarction, and stroke (12). We believe this study serves as guidance on assessing the care for diabetes at the national level in a minimal but sufficient way, particularly in countries with a similar context. It could also shed light on the likely scenarios of diabetic patients go through where the healthcare system resembles Iranian healthcare architecture.

## Materials and methods

### Overview

The current demonstration study is part of a nationally representative IQCAMP survey generating patient-centered real-world data on the utilization, costs, and quality of care for seven high-prevalence and high-cost diseases in Iran from 2016 to 2018. This study reports first-hand data on patient experience regarding healthcare utilization, costs, and quality of care for diabetes.

### Study protocol

The patients in the IQCAMP study were selected using a novel sampling method, the details of which are provided elsewhere (12). A machine-learning-based sampling method was used to divide the 31 provinces into eight clusters considering their similarity in healthcare structure and outcome data. One province from each cluster was selected for data collection. Simulation analysis of the sampling revealed an efficiency of up to 70% (12). In the selected clusters, patients with diabetes (13) were selected from the participants with diabetes from the STEPwise Approach to NCD Risk Factor Surveillance (STEPS) 2016 study, a national cross-sectional survey carried out by the Non-Communicable Diseases Research Center (NCDRC). Participants of the STEPS survey were selected *via* multistage cluster sampling, and they were representative of the general population aged ≥18 years living in urban and rural areas in all provinces of Iran. A detailed description of the study population and the sampling method of the STEPS survey has been published elsewhere (14). Diabetes was defined as the presence of fasting plasma glucose > 7 mmol/L or A1C > 6.5% or a past medical history of confirmed diabetes that is under treatment. All patients with diabetes who were aware of their disease were invited to participate in the study. Trained nurses called the patients and gave them detailed instructions on the study objectives and their right to leave the study at any time. All participants provided written informed consent. We collected the data through the phone. The initial interview included the participants’ current and past medical history. Then, three monthly follow-up interviews were held to collect information on service utilization, quality indicators, and the cost of healthcare services received. Tehran Medical Science University’s ethics committee and the National Institute of Medical Research Development (NIMAD) approved the patient recruitment protocol.

### Variable and data collection

The study assessed variables addressing utilization, quality, and costs of healthcare services. Healthcare services were categorized into three major groups: therapeutic, diagnostic, and patient support services. Therapeutic services included inpatient care, ambulatory care, and medication coverage. Lab and imaging services constituted diagnostic services. Rehabilitation could consist of healthcare services such as physiotherapy, occupational therapy, and speech therapy. Data were gathered from the health records of participants who underwent hospitalization and structured questionnaire-based interviews. Domain experts in epidemiology and endocrinology developed the initial draft of the study questionnaire, which patients with diabetes then debriefed. Finally, the study questionnaire was hosted on an android provisioned device and then went through usability testing for the study interviewers. The questionnaire consisted of four cardinal sections, including questions regarding participants’ sociodemographic and health status, frequencies of the utilization of various healthcare services, quality indicators, and healthcare costs.

The sociodemographic section included sex, age, literacy, and household wealth index. Principal Component Analysis (PCA) was applied to derive the household wealth index based on questions on key dwelling characteristics and household ownership, as described in the study protocol. PCA is an approach to statistical analysis in which multiple datasets are combined as orthogonal components (15). The wealth index was used to divide the population into quintiles, whereby the first and fifth quintiles present the least fortunate and wealthiest households, respectively. In the utilization section, participants were asked to declare frequencies of the utilization of any healthcare services throughout the study period. In addition, all medications available in the Iranian Pharmacopoeia were included in the survey.

A selection of pre-defined quality indicators was utilized to assess the quality of provided healthcare services. A panel of medical experts managing patients with diabetes considered different quality indicators using the guidelines of the Ministry of Health and Medical Services of Iran, frameworks utilized in developing countries, and the National Qualification Framework (NQF) designed for the United States (US) (16). The study’s expert panel added several essential indices based on their experience or literature review. The questionnaire was face-validated and then updated after a pilot study with the participation of ten patients.

Hospital invoices and patients’ out-of-pocket share were investigated to calculate the healthcare services’ costs. The related travel and accommodation expenses were added to calculate the direct cost of the disease. Lower income due to diabetes, loss of productivity, and wasted time of the patient and their possible accompanying family members during the doctor-patient appointments were calculated as indirect costs. Questions regarding healthcare diagnostic, therapeutic, and patient-support services were asked to help with out-of-pocket share estimations.

### Data analysis

The unit of analysis was defined as person-month. Annual average costs of diabetes were calculated by the average cost of each month multiplied by 12. Purchasing Power Parity (PPP) for 2018 was applied to convert Iranian Rials to US Dollars (USD), 1000 USD equaled 16,773,000 Iranian Rials (17). The number of absent days from work due to diabetes multiplied by the minimum daily wage was calculated the loss due to diabetes. We computed loss of productivity by asking the patients how much less they had earned in a month when they struggled with diabetes complications compared to a normal month. Diabetes care quality was assessed by reporting the percentage of participants meeting the defined quality standards. All quantitative data are reported by mean, standard deviation, number, and percent.

## Results

The final sample included 158 patients, among whom 91 (57.6%) were women. All patients underwent three successive monthly follow-ups, summing up to 474 patient months. The sociodemographic characteristics of participants are presented in **Table 1**.

**Table 1:** Sociodemographic characteristics of participants.

Total	159
Variable	N (%)
Sex	
Female	92 (57.8)
Male	67 (42.2)
Age group	
18-35 years	3 (1.9)
36-65 years	99 (62.7)
>65 years	45 (28.5)
Not defined	12 (4.9)
Education	
Illiterate	11 (7.0)
Primary school	42 (26.6)
Middle/high school	46 (29.1)
High school diploma	15 (9.5)
University graduate	22 (13.9)
Post-graduate degree	9 (5.7)
Not defined	13 (8.2)
Wealth index	
Very low	9 (5.7)
Low	47 (29.8)
Middle	38 (24.0)
High	39 (24.7)
Very high	25 (15.8)

### Healthcare utilization

The most utilized services were medication purchase, with times per month (159 times among women and 117 among men). The second most utilized type of healthcare service was outpatient visits with 231 times per month (137 times among women and 94 among men). **Table 2** presents the monthly utilization of various diabetes-related services among 474 visits.

**Table 2:** Monthly utilization of healthcare services among patients with diabetes.

Services		Patients	Events	Events per patient
**Therapeutic Services**				
Inpatient services	Total	9	11	0.04
	Women	7	8	0.04
	Men	2	2	0.02
Outpatient services	Total	160	231	0.74
	Women	96	137	0.74
	Men	64	94	0.74
Laboratory services	Total	79	85	0.27
	Women	54	60	0.32
	Men	25	25	0.2
Diagnosis services	Total	21	28	0.09
	Women	13	20	0.11
	Men	8	8	0.06
**Patient Support Services**				
Rehabilitation services	Total	3	3	0.01
	Women	3	3	0.02
	Men	0	0	0
Medication purchases	Total	218	276	0.88
	Women	125	159	0.86
	Men	93	117	0.92
Home care services	Total	2	2	0.01
	Women	2	2	0.01
	Men	0	0	0
Medical equipment	Total	27	27	0.09
	Women	14	14	0.08
	Men	13	13	0.1

### Healthcare quality

The mean (SD) time from the last laboratory blood sugar assessment was 4.44 (5.33) months, 3.5 (3.05) months among women, and 6.02 (6.51) months among men. While more than 90% of respondents showed a history of laboratory fasting blood glucose assessment in the previous year, less than 70% reported quarterly follow-up physician visits for diabetes management. During the follow-up visits in the last year, 93% of respondents reported that their blood pressure was measured during the office visit. However, less than half of the respondents reported weight measurement by any healthcare professional. While 75% of respondents reported receiving instructions on medication use from their consulting physicians, less than 50% said that pharmacy staff explained the medication use and timing.

Among 129 respondents, 9 (7.0%) had a history of ambulatory care due to hyper or hypoglycemia during the last year: 8 (10.4%) among women and 1 (1.9%) among men. An average of 3.71 (4.04) hypoglycemia episodes resulted in hospital admissions or outpatient visits in the previous year, 4.5 (5.2) among women and 2.67 (1.12) among men. Nevertheless, only 43% the respondents reported being interviewed for hypoglycemia episodes by their consulting physician during the last year. Notably, less than 45% of respondents reported receiving any training about hypoglycemia self-management from their physician or any other healthcare provider. The mean (SD) number of hyperglycemia episodes that have resulted in hospital admission or an outpatient visit in the previous year was 2.26 (1.5), 2.05 (1.4) among women, and 3 (1.7) among men. While 72% of respondents reported receiving medical advice on the significance of blood glucose control during the last follow-up visit, only 54% reported that their physicians had advised them to have more frequent follow-up visits for uncontrolled blood glucose.

Regarding lifestyle modification consults, around 70% of respondents received at least one episode of advice for regular physical activity and 62% for proper dietary habits. Only a quarter of respondents reported undergoing foot examinations by healthcare professionals, and less than 8% said that their shoes had been evaluated for diabetic foot prevention. Regarding diabetic foot prevention, less than 30% of respondents reported being trained for a regular self-foot examination and how to select the correct pair of shoes. Only 35% of respondents said they had received patient education materials for future reference. And finally, less than 10% of respondents reported receiving an influenza vaccine during the previous year, and 1.4% reported vaccination against pneumococcal infection during the last five years (**Table 3**).

**Table 3:** Diabetes healthcare quality indicators.

Indicator	Women		Men		Both	
	n (%)	Total	n (%)	Total	n (%)	Total
Comprehensive management						
Glycemic control						
Laboratory fasting blood glucose assessment in the last year	77 (86.5)	89	61 (95.3)	64	138 (90.2)	153
Over the past year, attended quarterly follow-up physician visits for diabetes management	54 (65.1)	83	45 (70.3)	64	99 (67.3)	147
As a result of uncontrolled blood sugar levels, doctors scheduled medical visits sooner than quarterly follow-ups	38 (54.3)	70	33 (54.1)	61	71 (54.2)	131
Weight measurement in the previous physician visit	40 (47.6)	84	31 (49.2)	63	71 (48.3)	147
Consulting physician asking about hypoglycemia episodes in the past year	38 (45.2)	84	27 (42.2)	64	65 (43.9)	148
Cardiovascular health						
Blood pressure measurement by a healthcare professional during the last year	81 (93.1)	87	62 (93.9)	66	143 (93.5)	153
Electrocardiography assessment during the last year	55 (63.2)	87	44 (67.7)	65	99 (65.1)	152
Lipid profile assessment during the last year	45 (49.5)	91	30 (45.5)	66	75 (47.8)	157
Microvascular complications						
Ever being referred for retinal examination	56 (62.2)	90	42 (62.7)	67	98 (62.4)	157
Feet examination by a healthcare professional regarding diabetic foot ulcer during the last year	23 (25)	92	17 (25.4)	67	40 (25.2)	159
Footwear evaluation by a healthcare professional during the last year	4 (4.6)	87	8 (12.5)	64	12 (7.9)	151
Mental health						
History of prescription of major depressive disorder medications	12 (85.7)	14	5 (71.4)	7	17 (81)	21
Being diagnosed with major depressive disorder by a psychiatrist	14 (15.9)	88	7 (10.8)	65	21 (13.7)	153
Infectious diseases prevention						
Vaccination against influenza during the last year	6 (7.1)	84	8 (12.3)	65	14 (9.4)	149
Vaccination against pneumococcal infection during the last five years	1 (1.2)	84	1 (1.6)	64	2 (1.4)	148
Dental health						
Referral to a dentist for gingival or dental assessment during the last six months	22 (25.6)	86	22 (33.8)	65	44 (29.1)	151
Patient education						
Treatment compliance						
Receiving medical consult on medication usage and intervals by consulting physicians	29 (82.9)	35	13 (61.9)	21	42 (75)	56
Receiving consult or medical consult on medication usage and timing by the pharmacy staff	19 (55.9)	34	8 (38.1)	21	27 (49.1)	55
Lifestyle promotion						
Healthcare professionals' recommendations on regular physical activity for blood sugar control in the past year	58 (63)	92	53 (79.1)	67	111 (69.8)	159
Healthcare professionals' recommendations on dietary habits for blood sugar control in the past year	58 (63)	92	41 (61.2)	67	99 (62.3)	159
Being advised to quit smoke by a healthcare professional during the last year	12 (13)	92	12 (17.9)	67	24 (15.1)	159
Selfcare promotion						
Medical advice on the significance of blood glucose control and the complications of diabetes during the last follow-up visit	67 (79.8)	84	39 (61.9)	63	106 (72.1)	147
Being trained about the measures to be taken upon hypoglycemia	34 (41)	83	32 (50)	64	66 (44.9)	147
Receiving patient education materials for future reference upon questions or concerns	30 (36.1)	83	22 (34.4)	64	52 (35.4)	147
Being trained about regular foot self-examination by healthcare professionals	22 (25)	88	21 (32.3)	65	43 (28.1)	153
Receiving a medical consult on the features of an optimized shoe	19 (21.8)	87	18 (28.1)	64	37 (24.5)	151

### Healthcare costs

**Table 4** conveys the direct and indirect costs of diabetes. The annual average direct health-related cost of a patient with diabetes was 768.96 USD. The average out-of-pocket share of direct health-related costs was 600.96 (78.15%) USD. Medication purchases, inpatient services, and outpatient services summed up 79.77% of direct health-related costs with a mean of 613.4 USD.

**Table 4:** Annual costs of medical services for patients with diabetes.

Services	Out-of-pocket costs		Total costs	
	Mean (SD)	Median (IQR)	Mean (SD)	Median (IQR)
Direct Costs				
*Health related*				
In-patient services	131.64 (17,806.20)	250.44 (250.32)	246.60 (16,905.84)	1,430.88 (15,782.40)
Out-patient services	83.16 (413.52)	143.04 (316.56)	139.56 (1,344.96)	243.24 (373.92)
Laboratory services	45.00 (337.32)	200.28 (317.64)	84.84 (1,008.48)	433.44 (816.96)
Diagnosis services	44.76 (1,244.16)	536.52 (1,171.68)	30.12 (999.48)	590.28 (1,799.40)
Rehabilitation services	4.44 (328.80)	697.56 (232.56)	4.44 (121.92)	700.80 (86.16)
Medication purchases	261.12 (769.32)	294.72 (416.76)	227.16 (925.80)	301.92 (354.24)
Home care services	3.48 (NA)	1,073.16 (0.00)	0.00 (NA)	NA (NA)
Medical equipment	27.36 (410.76)	400.68 (457.80)	36.24 (385.32)	400.68 (457.80)
Sum	600.96	N/A	768.96	N/A
*Non-health related*	N/A	N/A	325.08 (325.08)	214.68 (271.92)
Indirect Costs				
Lower income because of diabetes	N/A	N/A	0.84 (0.84)	0.60 (0.36)
Loss of productivity	N/A	N/A	2,978.52 (2,978.52)	2,146.32 (2,861.76)
Time waste	N/A	N/A	576.24 (576.24)	440.52 (630.48)
Sum	N/A	N/A	3,880.68	N/A

NA, Not applicable.

## Discussion

This study is the first nationally representative research that collects cost and quality information directly from patients at the national level. Medication purchases and outpatient medical visits were the most utilized healthcare services. Most direct costs were for medications and inpatient and outpatient services. Medication purchases and inpatient and outpatient services imposed the most significant proportion of out-of-pocket costs. The healthcare system's primary focus was on glycemic control rather than a fair distribution of services across preventive and therapeutic care according to standard guidelines for diabetes management (18).

Approximately 80% the direct health-related costs of diabetes were for medication purchases, inpatient services, and outpatient services. Similarly, it has been reported that inpatient and medication costs were the most expensive aspects of diabetes care in low- and middle-income countries (19). On average, out-of-pocket share constituted 78% of the total direct costs, primarily due to medication purchases. Evidence shows that change in out-of-pocket share for diabetes medications across various payer policies impacts diabetes medication usage. Patients sharing fewer drug payments tend to have a significantly higher number of months with apparent active medication coverage, a proxy for medication adherence (20).

Continuous medical care is required to achieve optimal glycemic control among patients with diabetes and prevent diabetes complications. The respondents reported receiving inpatient or outpatient healthcare services at least two times during the previous year. While home care could lead to improved diabetes-related outcomes among patients (21), it was among the least utilized services by respondents. It has been reported that some one-third of the inappropriate all-causes hospitalization stays in Iran were due to lack of home care, 35% of which was attributable to diabetes complications (22). Thus, establishing appropriate home care in the health system as well as covering home care expenses by insurance could optimize hospital bed use, reduce costs, decrease readmission rates, and prevent hospital-related complications.

More than half of the respondents said they were not asked about hypoglycemia episodes by their consulting physician during their routine follow-up visits in the last year. Based on patients’ reports, healthcare professionals did not train the patients in self-management of hypoglycemia episodes. Nevertheless, intensive antidiabetic treatments could impose patients at increased risk of hypoglycemia. While hypoglycemia-associated risk factors are yet to be adequately understood (23), the frequency and severity of hypoglycemia could be decreased *via* structured patient education (24). Telemedicine, as a novel and accessible tool, could be along with proper patient education to monitor blood glucose, thus reducing the risk of hypoglycemia (25).

Despite the clear benefit of weight loss in glycemic management (26), only a tiny percentage of patients with diabetes can maintain substantial weight loss (27). Notably, frequent follow-up visits can better achieve weight management (28). In our study, less than half of the respondents reported that they underwent weight measurement by healthcare professionals during the follow-up visits. However, 70% of respondents had been advised for regular physical activity and 60% for proper dietary habits.

Only a quarter of respondents reported undergoing foot examination by healthcare professionals, and less than one-tenth said their footwear had been evaluated for diabetic foot prevention. Moreover, less than one-third of respondents reported being trained in the regular self-foot examination and the features of an optimized shoe for patients with diabetes. The lifetime incidence of foot ulcers among patients with diabetes could be as high as 25%. There is strong supporting evidence for screening all patients with diabetes to identify those at risk for foot ulceration. High-risk patients could benefit from prophylactic interventions, such as patient education and prescription footwear (29).

Less than 10% of respondents reported receiving an influenza vaccine during the previous year, and only 1.4% said being vaccinated against pneumococcal infection during the last five years. Reasons for low utilization of vaccines among patients could be improper knowledge (30), lack of vaccine recommendations by physicians, mistrust of vaccine safety, inconvenience of vaccination procedure, supply, and accessibility (31).

Neglecting optimal long-term diabetes management can result in a higher prevalence of diabetes complications, reducing the patient’s quality of life and increasing healthcare expenditure. So far, there has been suboptimal diabetes management in the country, as reflected in poor glycemic control (32). Nevertheless, the issue is not specific to Iran, as less than 10% of patients with diabetes in low-income and middle-income countries receive guideline-based comprehensive diabetes treatment (33). Even in countries with well-established economies like the US, improving diabetes control at the national level is a new challenge (34). Part of this challenge is justified by the complicated nature of the patient-provider relationship in setting diabetes control goals when a patient visits the physician (35).

These concerns about diabetes care are a call for concerted efforts toward scaling up the capacity of healthcare systems to follow a complete, integrated care model for the management of diabetes, which provides patient-centered, holistic, and continuous healthcare services for patients with diabetes. Holistic approaches towards diabetes management could consist of multidisciplinary teams (36), close follow-ups (37), regular home visits (38), and medication review (39). Education should become an integral part of diabetes management to empower patients to take control of their disease. Telehealth technology could be utilized for continuous disease monitoring, delivering education materials, and lifestyle promotion as a novel approach. In particular, access to telehealth in addition to in-person visits can promote access to and use of diabetes care and consequently improve health outcomes and quality of life for people with diabetes (40).

The Middle East and North Africa (MENA) region is estimated to have the second-highest global growth rate in the number of affected individuals with diabetes (41). Since 2004, the National Program for Prevention and Control of Diabetes (NPPCD) of Iran has made endeavors toward diabetes prevention and sustained care for patients with diabetes (42, 43). Despite adequate access to core medications for diabetes care, significant heterogeneity remains in comprehensive diabetes management, especially in glycemic control and complications management (44). While 81% of cities in Iran could cover essential diabetes services, 19% could not provide even the lowest coverage level (45). Without a national system for integrated diabetes control, researchers have attempted to provide quality and cost measures of diabetes care with mostly indirect estimations (46–48).

## Strengths and limitations

This study presented a patient-centered disease-specific collection of insights on healthcare utilization, quality, and costs of diabetes in Iran. In developing countries, where integrated health record systems do not exist, such surveys usually consist of small samples from limited geographic areas. However, this study delivers information collected from a diverse geographic area of the country using a model-based clustering method to represent the country. The study’s follow-up modules confirmed the self-reported costs with the medical bills. Nevertheless, the small sample of this demonstration study hindered sound subgroup analyses statistically. While self-reports of service utilization and care quality guarantee the patient-centeredness nature of the responses, self-reports always suffer from various biases, such as recall. This study had a small but representative sample from the entire country. It successfully provided a frame of action and a methodological blueprint for a more extensive national-level study of the exact nature with a larger sample in the future, both in developing and developed countries.

## Conclusion

Healthcare services have focused on glycemic control, and the comprehensive management of diabetes is compromised by insufficient continuity of services for diabetes control. Medication purchases and outpatient medical services were the most utilized healthcare services among patients with diabetes. The most direct costs were medication purchases and inpatient and outpatient services. Medication purchases and inpatient and outpatient services imposed the most out-of-pocket costs.

## Data availability statement

The raw data supporting the conclusions of this article will be made available by the authors without undue reservation upon request.

## Ethics statement

The studies involving human participants were reviewed and approved by the Ethics Committee of the Tehran University of Medical Sciences. The patients/participants provided written informed consent to participate in this study.

## Author contributions

Conceptualization, SK and SSh. Data curation, FM, MM, NS, YF, SSe, FG, SRa, SK, and SSh. Formal analysis, MA-K, S-HG, NA, YF, MA, SRo, SSe, FG, and SSh. Funding acquisition, SSh. Investigation, MA-K, YF, SSe, NR, SK, and SSh. Methodology, FM, YF, MA, SSe, NR, SK, and SSh. Project administration, FM, YF, SSe, NR, and SSh. Resources, NR and SSh. Software, YF, MA, HZ, MKh, and FG. Supervision, YF, NR, and SSh. Validation, YF, NR, SK, and SSh. Writing – original draft preparation, MA-K, S-HG, and SSh. Writing – review and editing, MA-K, FM, S-HG, MM, NS, NA, YF, MA, SRo, HZ, MKh, SSe, MKe, FG, SRa, NR, SK, and SSh. All authors contributed to the article and approved the submitted version.
